# The role of oxytocin receptor gene (*OXTR*) DNA methylation (DNAm) in human social and emotional functioning: a systematic narrative review

**DOI:** 10.1186/s12888-018-1740-9

**Published:** 2018-05-29

**Authors:** Catherine Maud, Joanne Ryan, Jennifer E. McIntosh, Craig A. Olsson

**Affiliations:** 1 Deakin University Geelong, Centre for Social and Early Emotional Development, Faculty of Health, School of Psychology, 221 Burwood Highway, Burwood, VIC 3125 Australia; 2 Murdoch Children’s Research Institute, The Royal Children’s Hospital, Parkville, VIC 3052 Australia; 3 Department of Paediatrics, The University of Melbourne, Melbourne, VIC 3052 Australia; 4 Department of Epidemiology and Preventative Medicine, School of Public Health and Preventative Medicine, Monash University, Prahran, VIC 3004 Australia

**Keywords:** DNA methylation, Epigenetics, Human behaviour, Oxytocin gene, Oxytocin receptor gene

## Abstract

**Background:**

The neuropeptide Oxytocin (OXT) plays a central role in birthing, mother-infant bonding and a broad range of related social behaviours in mammals. More recently, interest has extended to epigenetic programming of genes involved in oxytocinergic neurotransmission. This review brings together early findings in a rapidly developing field of research, examining relationships between DNA methylation (DNAm) of the Oxytocin Receptor Gene (*OXTR)* and social and emotional behaviour in human populations.

**Method:**

A systematic search across Web of Knowledge/Science, Scopus, Medline and EMBASE captured all published studies prior to June 2017 examining the association between *OXTR* DNAm and human social and emotional outcomes. Search terms included ‘oxytocin gene’ or ‘oxytocin receptor gene’ and ‘epigenetics’ or ‘DNA methylation’. Any article with a focus on social and emotional functioning was then identified from this set by manual review.

**Results:**

Nineteen studies met eligibility criteria. There was considerable heterogeneity of study populations, tissue samples, instrumentation, measurement, and *OXTR* site foci. Only three studies examined functional consequences of *OXTR* DNAm on gene expression and protein synthesis. Increases in *OXTR* DNAm were associated with callous-unemotional traits in youth, social cognitive deficits in Autistic Spectrum Disorder (ASD), rigid thinking in anorexia nervosa, affect regulation problems, and problems with facial and emotional recognition. In contrast, reductions in DNAm were associated with perinatal stress, postnatal depression, social anxiety and autism in children.

**Conclusions:**

Consistent with an emerging field of inquiry, there is not yet sufficient evidence to draw conclusions about the role of *OXTR* DNAm in human social and emotional behaviour. However, taken together, findings point to increased *OXTR* DNAm in general impairments in social, cognitive and emotional functioning, and decreased *OXTR* DNAm in specific patterns of impairment related to mood and anxiety disorders (but not in all). Future progress in this field would be enhanced by adequately powered designs, greater phenotypic precision, and methodological improvements including longitudinal studies with multiple time-points to facilitate causal inference.

## Background

Oxytocin (OXT) is a neuropeptide and hormone implicated in prosocial human social and emotional functioning [1]. OXT has been long known for its key role in child birth and postpartum lactation, including changes in infant gut hormones [2]. OXT plays a crucial role in enhancing bonding and moderating the stress response. Mothers’ neuroendocrine response to their baby’s cry, as measured by functional neuroimaging (fMRI), has shown to differ between vaginal and caesarean births, with higher OXT levels linked to enhanced empathy and maternal arousal [3]. Mothers with higher plasma OXT levels during early pregnancy have been shown to be more responsive to their baby after birth, including higher levels of gaze towards their infant’s face, positive affect and affectionate touch [4].

Postpartum, the sucking stimulus by newborns increases OXT release in the mother and thus decreases plasma levels of stress hormones (ACTH and cortisol) [5]. The physiological consequences of disrupted OXT release are thus significant during the perinatal period, however, there is also mounting evidence that disrupted OXT availability could be related to a broad range of social behaviours, including childhood autistic spectrum disorders (ASD) [6], attachment bonds in infancy [7] and mood disorders and response to stress in women [8].

This has led to the development of OXT treatments including using nasal OXT to increase attachment security in healthy males with an insecure attachment pattern [9] and improved social engagement between fathers and their infants [10]. Further, intranasal OXT increased trust and willingness to accept social risks amongst healthy volunteers involved in an experimental game [11], as well as the reduction of the short-term stress response demonstrated by reduced cortisol release [12]. In patients with Autistic spectrum disorder (ASD), intranasal OXT has been shown to provide some improvements in adult social cognition [6] and youth emotional recognition [13].

fMRI studies have shown that during a visual stress task, intranasal OXT administration is associated with reduced activation in the amygdala, which plays a key role in emotion and social behaviour regulation [14]. Furthermore, central OXT has both anxiolytic and antidepressant effects which have been associated with improved emotional behaviours and mental health [15]. This has led to the suggestion that OXT may be an important biomarker of stress exposures [16]. OXT plasma concentrations also correlate with cerebrospinal concentrations and with childhood anxiety [17]. Disrupted peripheral OXT release has been found in non-perinatal women with depression during stress related tasks [8] and with specific types of relationship stress in women [18]. Taken together, these findings suggest an important role for OXT in human social and emotional functioning.

Neurobiologically, OXT is synthesized by the OXT gene in magnocellular neurons in the paraventricular and supraoptic nuclei of the hypothalamus. OXT is then transported along axonal projections to the posterior lobe of the pituitary, where it is stored in secretory vesicles before being released into the peripheral blood. In addition, dendritic release of OXT occurs into the extracellular space with diffusion through the brain. Smaller parvocellular neurons in the paraventricular nucleus also produce OXT and project directly to other regions in the brain involved in human social and emotional functioning including the amygdala, hippocampus, striatum, suprachiasmatic nucleus, bed nucleus of stria terminalis and brainstem - where they act as neuromodulators or neurotransmitters and thereby influence neurotransmission in these areas [19].

Oxytocin receptors are synthesised by the Oxytocin Receptor (*OXTR*) gene, which expresses both centrally in the brain and within peripheral organs. In this way, OXT has both peripheral and central functions. *OXTR* is located on human chromosome 3p25.3 [20, 21]. The *OXTR* gene spans 17 kilobytes (kb) and contains 3 introns and 4 exons. Exons 1 and 2 correspond to non-coding regions while exons 3 and 4 encode the amino acids of the oxytocin receptor (Fig. 2). The oxytocin receptor is a 389-amino acid G-protein coupled transmembrane receptor, enabling the activation of a number of different intracellular secondary messengers facilitating the oxytocin pathway [22].

Genetic variants, including single nucleotide polymorphisms (SNPs), in the *OXTR* have been shown to independently influence gene expression and been associated with human emotional responsiveness and social behaviour [23]. Two SNPs *rs2254298* and *rs53576* have been most investigated regarding the *OXTR* (Intron 3) (Fig. 2). *Rs2254298* (G > A) has also been associated with autistic spectrum disorder (ASD) [24], depressed women and their families [25] and with smaller bilateral amygdala volumes and greater grey matter volumes in a brain imaging study [26], while *rs237887* has been associated with human social recognition skills [27]. *Rs53576* (A > G) has been associated with reduced positivity [28], reduced empathy [29] and decreased parental sensitivity [30] while *rs1042778* has been associated with reduced parenting sensitivity [31] and *rs237915* has been associated with brain responsiveness to social cues [32]. A meta-analysis of 11 independent populations of patients with ASD reported associations with *rs7632287, rs237887, rs2268491* and *rs2254298* [33].

The focus of this review is on the newly emerging field of *OXTR* epigenetics and socio-emotional functioning in humans. Epigenetic mechanisms are reversible modifications occurring “above” the level of the DNA sequence, which can influence gene expression [34]. The most widely investigated and understood epigenetic mechanism is DNA methylation (DNAm), which commonly involves the addition of methyl groups to the cytosine and guanine dinucleotides termed “CpG” dinucleotides in the DNA sequence. *OXTR* is of particular interest because, unlike OXT, which has tight regional expression, *OXTR* is expressed extensively through brain and body and can thus control the final (rate-limiting) step in signal transmission. Differential methylation of the *OXTR* has been associated with changes in *OXTR* expression both in animals and in humans.

In mouse models, *OXTR* DNAm patterns have been correlated with differential *OXTR* expression in different body tissues [35]. In different brain areas, differing DNAm at specific CpG regions within the *OXTR* occurred with differential expression of *OXTR* [36]. The differential *OXTR* expression is considered to relate to a diversity of animal social behaviours arising from the limbic brain structures. Though animal and human *OXTR* gene promoter brain regions differ, the inference is that DNAm differences result in *OXTR* transcription changes with impact on human social and emotional behaviour.

In human samples, a region within the *OXTR* third intron between regions encoding trans-membrane domains 6 and 7, has been shown to be hyper-methylated within non-expressing tissues, but hypo-methylated in the uterine myometrium at term, when *OXT* is up-regulated for the birthing process [21]. Further work on *OXTR* has identified a region between the first exon and the first intron (MT2 segment) that is linked to tissue specific expression *OXTR* in peripheral blood mononuclear cells (PBMCs), liver, and uterine myometrium (from non-pregnant and term-pregnancies) [37]. Genetic variation may also influence patterns of DNAm within the gene [38–41] and thus can have both combined and independent effects on gene expression [42, 43]. However, lack of replication to date requires cautious interpretation.

The purpose of this review by narrative synthesis is to bring together the emerging evidence on *OXTR* DNAm and human socio-emotional functioning. The diversity of studies available in this new area of research currently precludes meta-analytic presentation of findings. This review provides a timely summary of a new field of research around *OXTR* and socio-emotional behaviour, with implications for future promotion of emotional health in both population and clinical settings.

## Method

A review of the literature was undertaken to identify all published studies that investigated oxytocin receptor gene *(OXTR)* DNAm in human social behaviour, with a focus on social and/or emotional functioning. We defined human social and emotional functioning as the experience, expression and management of emotions as well as the ability to establish positive and rewarding relationships with others [44]. This extends to relationships, response to stress and social cognition with a basis in neurobiology, which has become more available to research through functional brain scanning.

### Search strategy and inclusion criteria

Included studies were identified using review methodology, conducted across four primary search database platforms: Web of Knowledge/Science, Scopus, Medline and EMBASE and included papers through to May 2017. The search terms used were: ‘oxytocin gene’ or ‘oxytocin receptor gene’ and ‘epigenetics’ or ‘DNA methylation’ and limited to studies involving humans and those written in English. Four different searches were completed with each search using terms: ‘oxytocin gene’ or oxytocin ‘receptor gene’ and one of ‘epigenetics’ or ‘DNA methylation’. These four different term search strategies were repeated from October 2013 to May 2017 on three separate occasions. This allowed for identification of new research as well as the confirmation of those articles already located. There were no exclusions based on participant age or study design. Studies which measured *OXTR* DNAm in the context of oncology, pregnancy or lactation were also excluded. The search was not restricted to specific psychosocial terms; rather, articles with a focus on emotional and social functioning were identified by manual review from the full set meeting criteria for epigenetic search terms. Manual identification of studies was also employed by examining the reference lists of original research articles retrieved, as well as reviews published in this area. No new articles were identified by this latter method that had not already been retrieved from the original designated term searches.

From all studies identified through the initial search, titles and abstracts were examined to determine their suitability for inclusion in the review. Titles often identified the article related to non-human studies, focused on oncology/obstetrics, related to oxytocin or not to epigenetics. When uncertainty occurred, further investigation of the abstract clarified the exact contact of the article. Studies included were original research articles investigating epigenetic regulation of *OXTR* and its association with social or emotional behaviours in humans, including social perception, mood problems (depression and stress), behaviour problems (eating disorder and callousness) and rarer neurodevelopment disorders (autism). No epigenetic studies were found where mechanisms other than DNAm were considered.

## Results

**Fig. 1:**
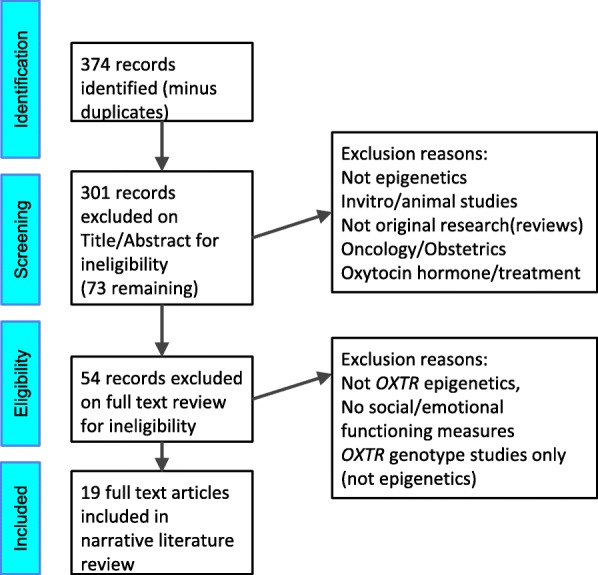
PRISMA Flow Diagram

**Fig. 2:**
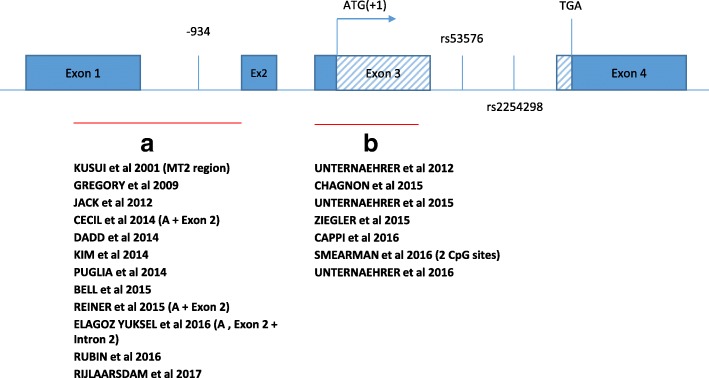
Oxytocin Receptor Gene (*OXTR* Chr:3 p25)

Figure 1 presents a PRISMA flow diagram showing initial study identification and stepwise exclusions of ineligible studies. A total 374 articles were initially identified for review. Of these a total of 19 were eligible for inclusion in the final review. Details of each are provided in Table 1. Study populations varied widely in age, heritage and from general population to clinical samples. Sample sizes were variable and predominantly small though size has been increasing in more recent research [45]. Most studies focused on the “MT2 segment” (between Exon 1 and Intron 1), the region identified as functionally significant in the initial gene expression studies (Fig. 2, Area A) [21, 37, 46]. Increased DNAm of this region, including the site − 934, has been linked to decreased *OXTR* gene transcription in both PBMCs and brain [46]. No study has examined the extent to which DNAm influences gene expression in Exon 3, (Fig. 2, Area B).

**Table 1:** Studies investigating the association between DNA methylation (DNAm) of the oxytocin receptor (*OXTR*) gene and human behaviour (by year of publication)

Study	Characteristics	Main Measure	Tissue	*OXTR* region & measurement	Methylation Association	Adjustment
Gregory et al. (2009) [46]	(i). 119 Autistic Spectrum Disorder (ASD) probands (78% male) & families. (ii). 20 ASD (50% male) & 20 controls. (iii). 16 brain samples in 8 ASD (75% male) & 8 controls (Caucasian US)	ASD (DSM-IV criteria) & ADI-R	PMCS & brain tissue (temporal cortex)	2 regions: (i) exons 1, 2 & 3 incl. MT2 ^a^ (ii). 3rd intron No SNPs DNAm Specific sequencing	i) ASD Family study: ↑ DNAm 5/22 CpG sites (− 860, − 901, − 924, − 934 & -959) in region (i) with significant associations at − 860 & - 934 in males & -959 in females ii) ASD vs controls: ↑DNAm 3 CpG sites (− 860, − 934, − 959). iii) Brain samples: ↑ DNAm 4 CpG sites (− 860, − 901, − 924, − 934) iv) ↓ Gene expression: PCR ↓mRNA in ASD male brains	Matched: age & gender.
Jack et al. (2012) [42]	42 healthy adults (55% male) 18–30 yrs. Inclusion: normal vision & no previous psychiatry (67%Caucasian US)	fMRI brain scan of during visual perception animation tasks.	PMCS	- 934 single site No SNPs Pyrosequencing	↑ DNAm site −934 associated with ↑fMRI activity in temporo-parietal junction incl. dACC during tasks.	No racial or gender differences
Unternaehr-er et al. (2012) [48]	76 adults (43% male) 61–67 yrs. (Caucasian Germany).	TSST	3× whole venous blood: before TSST, 10 & 90 mins	2 regions in exon 3: *OXTR*1 & *OXTR*2 No SNPs Sequenom EpiTYPER	↑DNAm 7/8 CpG sites *OXTR*1: pre to 10 mins post-test & ↓DNAm from 10 mins to 90 mins post-test. ↓DNAm trend 2/27 CPG sites *OXTR*2: 10 to 90 mins. Post-test.	Blood cell counts & batch effects. No sex differences. Multilevel models
Cecil et al. (2014) [53]	84 youth with conduct disorder^b^ (no gender ratio), (UK).	High Callous Unemotional (CU) traits maternal assessed at 13 yrs	PMCS in cord blood, 7 & 9 years	12 probes across gene. SNPs:4 CU focus Illumina array 450 K^c^	↑DNAm 3/12 probes on exon 2 in cord blood with ↑ CU in low internalising problems group & with ↑parental risk exposures (prenatal & late-childhood. SNPs *rs2301261, rs237915, rs4564970, rs4686302* NS^d^ with DNAm, CU or internalising groups.	Gender stratified analysis (internalising problems)
Dadds et al. (2014) [49]	98 male; 4–16 yrs.: conduct disorder or Oppositional Defiant Disorder (ODD) Exclusion: ASD, neurological /physical illness, ↓IQ. (Australia).	i).ODD /Conduct Disorder assessment^e^ ii). CU traits^f^	Blood	11 probes: exon 1 - intron 1. No SNPs Sequenom MassARRAY	Children 9-16 yrs., ↑ DNAm associated with ↑ CU traits. Examining the 6 individual CpG sites, these findings were predominantly with CpG 5 Subset 37 youth ↑DNAm associated with ↓ circulating OXT protein levels, in older children only.	Covariates tested.
Kim et al. (2014) [39]	51 females, 18 + yrs. (15 Anorexia Nervosa (AN); 36 healthy controls (South Korea) Exclusion: ASD, psychosis & substance use disorder.	SCID (DSM-IV) & EDE-Q, BMI, AQ (ASD traits), DBI & STAI.	Buccal	48 probes: exon 1-intron 1. No SNPs DNAm specific sequencing	↑DNAm 5/48 CpG sites & averaged across region associated with AN. ↓BMI correlated ↑DNAm at 5/48 CpG sites ASD traits did not associate with DNAm	Age, BMI & clinical variables (ASD, anxiety & depression)
Bell et al. (2015) [34]	269 female postnatal depression (131 antenatal depression); 276 matched controls (135 antenatal depression). 2/3 s: 25–34 yrs. (Caucasian UK)	EDPS: antenatal & postnatal	Whole antenatal blood	- 934 single site SNPs *rs53576* & *rs2254298* Pyrosequencing	↑DNAm for females with postnatal depression only in *rs53576* GG	Matched: age, parity, depress-ion. Adjusted Models: psychosocial covariates.
Chagnon et al. (2015) [33]	43 females > 65 yrs. (18 prev. Anxiety & MDD, 1 with MDD; 24 controls. Excluded: psychosis, schizophrenia or MMSE score < 22. (Canada).	Anxiety & MDD (DSM-IVR)^g^	Saliva	9 probes in 2 regions: exon 3. SNPs: *rs53576* Pyrosequencing	No difference between groups overall. ↑DNAm (× 2) for females with anxiety/depression who were *rs53576* AA when averaged across region 1 (4 probes) and for 1/5 CpG sites of region 2.	Matched: no details
Puglia et al. (2015) [47]	98 healthy adults, (43% male) 18–30 yrs.: normal vision. (Caucasian, US)	fMRI brain scan during emotional face matching task	PMCS	- 934 single site No SNPs Pyrosequencing	↑ DNAm associated with fMRI responses: ↑ face & emotion processing areas (amygdala, fusiform & insula) & ↓ connectivity between social perception systems & ↓ coupling amygdala & emotional regulation regions.	Gender stratified analysis. Age: NS
Reiner et al. (2015) [35]	85 premenopausal female adults (43 depressed & 42 controls), aged 19–52 yrs. Excluded: psychosis, medical, personality, eating & substance use disorders. (Caucasian Germany).	MDD (SCID-DSMIV)	PMCS	Mean DNAm: exon 1(36 sites); exon 2(6 sites) SNP: *rs53576* DNAm Specific sequencing (ESME software)	↓DNAm in exon I (not exon 2) in depressed females. ↓DNAm in depressed females rs53576 GG > AG/AA but in healthy controls ↑DNAm in GG > AG/AA. ↓DNAm in exon 2 associated with *rs53576* GG & not depression	Matched: age and education. Mixed models: age, alcohol, smoking, BMI antidepressant use.
Unternaehr-er et al. (2015) [52]	85 university students with maternal care: low (45) and high (40) (79% female) aged 19–66 (median 24). (Caucasian, Switzerland)	PBI (maternal care) when < 16 years: retrospective assessment	Whole blood	2 regions in exon 3 (*OXTR*_*TS1*_ & *OXTR*_*TS2*_). (35 CpG units) No SNPs Sequenom EpiTYPER	↑ DNAm in low versus high maternal care in *OXTR*_*TS2*_ ↓DNAm in males compared to females independent of maternal care in *OXTR*_*TS2*_.	Adjusted: age, gender, batch effects. Mixed model analysis: cell count, BMI & depression considered.
Ziegler et al. (2015) [51]	220 adults: 110 un-medicated social anxiety (76 female & 34 male); 110 controls (77 female & 33 male) Excluded: medical, psychosis & substance use disorders. (Caucasian Germany)	Psychological: SIAS, SPS; cortisol response to TSST, fMRI of amygdala during social anxiety word task.	Whole blood	Region in exon 3 with 12 CpG sites. SNP: *rs53576* DNAm Specific sequencing (ESME software)	↓DNAm at 1/12 CpG site with psychological measures, cortisol response & fMRI. ↓average DNAm across all 12 CpG sites in social anxiety patients compared to controls. ↓average DNAm & at 7/12 CPG sites for rs53576 AA/AG > GG.	Matched: age and sex
Cappi et al. (2016) [40]	73 adults: 42 OCD (9 CBT or fluoxetine /33 treatment naïve); 31 controls: 18–65 yrs. Excluded: psychosis, suicide risk, prev. Head injury, medical & substance use disorders (Brazil)	OCD(DSMIV): Y-BOCS score ≥ 16 (obsessions & compulsions) or ≥ 10 (obsessions or compulsions), BDI, BAI, YGTTS.	Peripheral blood leucocyte ≤2 weeks treatment	2 regions exon 3:*OXTR 1* (4 CpG sites) & *OXTR 2* (5 CpG sites). No SNPs Pyrosequencing	↑DNAm at 2/9 CpG sites in OCD > controls. ↑DNAm correlated with ↑OCD severity ↑DNAm at 8/9 CpG sites with ↓BDI score. No difference DNAm between 33 treatment naïve or 9 fluoxetine/CBT. No DNAm correlation between OCD & tics.	Examined age, sex, education, BDI score, BAI score, tics, YBOCS score
Elagoz Yuksel et al. (2016) [41]	66 children: 27 ASD (23 male & 4 female); 39 controls (6 female & 33 male), 22–94 months. Excluded: medication, ↓development, neuro-degenerative, medical & psychiatric disorders(Turkey)	ASD (DSMIV-TR) CARS (≥30 for ASD).	Peripheral blood	4 regions: exon 1 – exon 3 (MT 1–4). No SNPs DNAm Specific sequencing	↓DNAm in 2/4 regions: MT1 & MT3 in ASD > controls. MT1 site (exon 1) DNAm %: 44.4% ASD & 71.8% healthy controls MT3 site (intron 1, exon 2, intron 2) DNAm %: 29.6% ASD & 61.5% healthy controls.	Matched: age & gender.
Kimmel et al. (2016) [43]	51 females postnatal depression: average 30.6 yrs. (70% Caucasian). Previous mood disorder (66% MDD, 33% Bipolar Disorder) (US)	MDD (DSMIV) ≤ 1st month >birth.	Whole blood T3^h^	18 CpGs sites SNP: *rs53576* Illumina array 450 K (Brain Cloud tool)	*↓*DNAm 1/18 CpG site in female postnatal depression. *rs53576* NS	Adjusted cell proportions.
Rubin et al. (2016) [50]	242 adults: 167 with psychosis (affective/bipolar disorder & non-affective/schizophrenia (92 female & 75 male); 75 healthy controls (38 female & 37 male). Excluded: previous head injury, ↓ reading, medical & substance use disorders (US)	Psychosis (DSMIV); Penn Emotional Recognition Task; fMRI during facial recognition task (79% sample)	Whole blood	A single CpG site (−934) No SNPs Pyrosequencing	↑DNAm schizophrenia > bipolar disorder ↑DNAm psychotic females >males ↑DNAm with ↓emotional recognition in females & controls > males. ↑DNAm with ↓ brain volumes: temporal-limbic and prefrontal regions (social cognition) in healthy controls & schizophrenia females. Plasma OXT levels and DNAm NS in psychotic/ controls Gender differences in plasma OXT & DNAm: (−) males & (+) for females.	Age, race, intracranial brain volume Sex stratified.
Smearman et al. (2016) [36]	393 adults (70.7% female) 18–77 yrs.: childhood abuse & current anxiety/depression (African American, US).	CTQ, HAMA, BDI Traumatic Events Inventory	Whole blood	18 CpG sites (before exon 1-intron 3) SNPs 44 Illumina array 450 K	↑ DNAm associated childhood abuse at 2/18 CpG sites but NS. DNAm not a mediator of psychiatric symptoms. 44 proximal SNPs: 68% associated DNAm of nearby CPG sites.	Age, sex & cell type
Unternaehr-er et al. (2016) [44]	39 infants with mothers mean age 31.9 yrs. (no sex ratio)(Switzerland)	Pregnant mother: EDPS, TICS-K, ILE, saliva cortisol in T2^i^, T3^h^.	Cord blood	13 CpG sites (exon 3) No SNPs Sequenom EpiTYPER	↓ DNAm associated with ILE, EDPS, T2 maternal cortisol (AUCg).	Batch effects, demographics, pregnancy & births.
Rijlaarsdam et al. (2017) [45]	743 children (51% male) ≤6 years yrs. (Netherlands)	ASD 6 yrs. with parental ratings: SRS. Maternal prenatal stress	Cord blood	3 CpG sites across gene. SNP*: rs53576* Illumina array 450 K	DNAm & prenatal maternal stress or ASD: NS ↑DNAm associated ↓social communication: *rs53576* GG	Child sex, age, cell type, maternal smoking & technical array.

a. MT2 segment is a genomic region on *OXTR* identified by Kusui, 2001; b. Diagnostic Interview Schedule for Children, Adolescents & Parents; c. 450 K: Illumina Human Methylation 450 Bead Chip array (Illumina, USA); d. Not Significant; e. Antisocial Process Screening Device/ASPD & pro-social subscale of Strengths & Difficulties/ PSSSDQ; f. Youth with early onset & persistent conduct problems assessed by Strengths & Difficulties Questionnaire ‘Conduct problem’ subscale; g. Diagnostic Interview Schedule and Composite International Diagnostic Interview for anxiety and depressive disorders; h. pregnancy second Trimester; i. pregnancy third Trimester

Only one of the 19 studies directly examined the relationship between *OXTR* DNAm and gene expression [46]. Two studies investigated a possible association between DNAm changes at the *OXTR* and subsequent oxytocin neuropeptide synthesis [47, 48]. As DNAm marks can be cell and tissue specific, it is important to note that most studies have used DNA derived from blood samples. One study each used buccal [49] and saliva samples [38], the latter of which may contain buccal epithelium and blood cells, including peripheral blood mononuclear cells (PBMCs). A third study investigated a small subset of brain tissue collected post-mortem [46].

### Social perception

Jack et al. [50] and Puglia et al. [51] examined the association between *OXTR* DNAm and brain activation using fMRI brain scanning while participants completed social perception tasks. The first involved visual animation tasks with different social perceptions [50], while the second used emotional face-matching tasks [47]. Both studies involved healthy young adults and examined *OXTR* DNAm in PBMCs, focusing on only one specific CpG site (− 934), around the MT2 segment. In both studies, results showed DNAm to be positively associated with brain activity in specific regions. These included the temporal parietal junction and dorsal anterior cingulate brain areas, regions associated with the face, the amygdala and fusiform gyrus and insula involved in emotion processing. Increased DNAm was also associated with decreased connectivity between the neural systems supporting social perception and decreased functional coupling of the amygdala within regions associated with affect appraisal and emotional regulation.

### Social stress

Unternaehrer et al. [52] examined the possibility of dynamically changing levels of DNA methylation in response to a social stress test within a longitudinal study design. DNAm at two proximal regions of the *OXTR* promoter was measured in whole venous blood over a 90-min interval, in 75 older individuals. In one of the regions, an increase in DNAm at the majority of CpG sites was found from pre-test to 10 min post-test. At both *OXTR* regions DNAm decreased between 10 min post-test and 90 min follow-up. The effect sizes observed were small but significant. The investigators completed cell counts and controlled for these differences in their analysis, also acknowledging that changes in dynamic cell composition could contribute to their results.

### Anxiety disorders

Two studies examining *OXTR* DNAm in the exon 3 region from blood and anxiety disorders were identified [53, 54]. Ziegler et al., investigated 110 adults with social anxiety compared to 110 healthy controls and noted reduced DNAm at only one of twelve CpG *OXTR* exon 3 sites on psychological, cortisol response and fMRI measures [54]. When average DNAm was considered across all twelve CpG sites, reduced methylation was also observed compared to controls. No difference between socially anxious patients and controls was noted when the *rs53576* genotype was investigated. Furthermore, Cappi et al., studied 73 adults with obsessive compulsive disorder (OCD) compared to 31 healthy controls and found increased DNAm at two of nine CpG *OXTR* sites in OCD adults compared to controls [53]. DNAm was positively correlated with OCD severity. In contrast, an inverse correlation was found between DNAm at eight of the nine sites for depressive scores in OCD adults. This could suggest that social anxiety and OCD sit on different dimensions, as is now the case in the latest iteration of the DSM-5 in which OCD is considered to be related to ASD and rigidity of thinking where as social anxiety disorder is considered a form of mood disorder.

### Depression

Reiner et al. compared *OXTR* DNAm in PBMCs between depressed pre-menopausal women and non-depressed age-matched controls [40]. Three fragments across exons 1 and 2 of *OXTR* (A, B and C) were measured. A decrease in DNAm of the exon 1 fragment A, (Fig. 2) was observed in depressed compared to non-depressed women. A specific *OXTR* SNP *rs53576* with a G to A allele change, known to be associated with reduced pro-sociality and decreased empathy, was also investigated [29]. This SNP moderated the association between DNAm of exon 1 and depression status and was found to be independently associated with exon 2 methylation levels. Depressed individuals with the A allele had increased mean DNAm compared to those homozygous for the G allele but healthy controls had the opposite result [40].

A further study by Chagnon et al. compared 19 depressed older women (all but one of whom also had anxiety) with 24 controls both in terms of saliva DNAm, average methylation across exon 3, and with regard to *rs53576* [38]. There were no differences in overall DNAm between groups; however, depressed women homozygous for the A allele of *rs53576* showed a significant increase in DNAm compared to controls. Different genotypes may have an independent impact on DNAm, which could contribute to these differing results [23]. The age of the population, including physiological states such as weight loss, hormonal and mood influences and the independent effect of the genotype itself may variously account for the different results.

### Perinatal disorders

Unternaehrer et al. also investigated *OXTR* DNAm in the cord blood of 39 infants born to mothers who had completed psychological testing and saliva cortisol testing during the second and third trimesters of their pregnancies [55]. Cord blood is the first available tissue following birth, which allows for the exclusion of postnatal factors impacting on the DNAm process. In their final testing 7 *OXTR* CpG units, including 13 sites, were investigated for DNAm with varying results. Reduced DNAm in the cord blood of the infants was associated with the total number of stressful life events in the two years prior to the second trimester, but not the strain caused by these events. Likewise, reduced DNAm in infant cord blood was associated with maternal depressive symptoms in the second trimester and certain elevated salivary cortisol level measurements from the second and third trimesters. The investigators used specific mixed modelling and batch effects analysis to obtain more reliable results.

Using data from the Avon Longitudinal Study of Parents and Children (ALSPAC), Bell et al. measured *OXTR* DNAm in whole blood, as well as two *OXTR* SNPs *rs53576* and *rs2254298* and investigated associations with perinatal depressive symptoms [39]. While no main effects were observed, overall a significant DNAm by genotype interaction was associated with the risk of postnatal depression. For women who were *rs53576* GG homozygous alleles, there was a positive correlation between *OXTR* DNAm and depressive symptoms postpartum. This finding is in contrast to the other studies of depression where associations were found between *OXTR* DNAm and depression risk for individuals with the *rs53576* A allele [38, 40], possibly accounted for by hormonal differences compared to mood disorders at differing times in the life-cycle.

Additionally, Kimmel et al., investigated 51 women with postnatal depression considering the complex hormonal impact, including estradiol on *OXTR* methylation [56]. Though 18 *OXTR* loci were investigated, only one demonstrated significantly decreased DNAm in the whole blood samples of the cohort and no significant *rs53576* analysis was noted.

### Maternal care

In a sample of 85 university students, Unternaehrer et al. compared *OXTR* DNAm across groups who retrospectively reported low or high maternal care [57]. No SNP genotypes were examined. Students reporting a history of low maternal care had increased DNAm at one of 23 CpG units examined. In addition to the retrospective self-reporting of early maternal care, confounding from other factors is an obvious limitation given the large time gap between childhood and the period when DNAm was measured. Smearman et al. likewise examined association between *OXTR* and retrospective reporting of childhood abuse and adult psychiatric symptoms (anxiety and depression) [41]. This study, with 393 African American participants, involved a comprehensive investigation of 18 CpG sites across the *OXTR* gene, as well as 44 *OXTR* SNPs. Of the 44 SNPs examined, 68% were associated with proximal DNAm levels. Non-significant trends were observed between childhood abuse and higher DNAm at two CpG sites in exon 3, as well as an interaction between childhood abuse and DNAm to predict adult psychopathology. Six SNPs of interest were also shown to interact with childhood abuse in determining risk for psychiatric symptoms, independently of DNAm levels.

### Anorexia nervosa

Kim et al. studied Korean patients with Anorexia Nervosa and investigated DNAm of the *OXTR* MT2 region in buccal samples [49]. They found increased DNAm in 15 clinical patients compared to 36 University recruited controls. The extent of DNAm negatively correlated with illness severity (BMI) and eating disorder psychopathology. As no longitudinal analysis was undertaken, the temporal relationship between increased DNAm and onset of Anorexia Nervosa could not be established. It remains unclear whether changes in *OXTR* DNAm patterns precede or are the consequence of the disease.

### Callous unemotional

Two studies measured peripheral blood *OXTR* DNAm in children and adolescents with conduct problems, with a particular focus on Callous Unemotional (CU) traits [47, 58]. As part of the ALSPAC study, Cecil and colleagues measured *OXTR* DNAm at birth in a subset of 84 boys and girls and found a positive association with CU traits in later childhood (7–9 years) [58]. There was no difference in association when the groups were stratified by internalising problems. In contrast, Dadds et al. focused on males only (*n* = 98) and found no association between *OXTR* promoter DNAm and CU traits overall, but some evidence that increased DNAm levels at one particular CpG site were associated with increased CU traits in older youth (aged 9–16 years) [47]. In a small subset of this cohort (*n* = 37), authors reported increased DNAm and lower oxytocin protein levels in the blood of older children (aged 9–16 years) but caution against over interpretation due to power limitations. Additionally, 9 *OXTR* SNPs were examined. Only *rs1042778* replicated an association with high CU traits across sexes and between childhood versus adolescent age groups within 2 separated geographical groups [59].

### Autism Spectrum disorder

While numerous studies have investigated the role of *OXTR* genetic variations in ASD, only three to date have examined *OXTR* DNAm [45, 46, 60]. The first study by Gregory et al., reported findings from three separate analyses [46]. The first included screening of 119 ASD pro-bands and their families to identify DNAm differences at *OXTR* sites in PBMCs, focusing on the area in the promoter region around the MT2 site. Increased DNAm was noted in ASD pro-bands compared to healthy family members. This analysis was complemented by a small ASD case-control study (*n* = 40) focusing on the same *OXTR* sites identified in their initial analysis demonstrating similar results. The authors also used eight samples from a brain bank of patients with autism and eight controls and compared DNAm levels in PBMCs with these brain temporal cortical samples, finding both had increased DNAm. This group further examined brain *OXTR* DNAm with gene expression in samples of temporal cortex from 4 ASD pro-bands (3 males and 1 female), matched with controls. For the 3 males only, *OXTR* expression was reduced by 20% compared to controls. Results from these studies suggest remarkable consistency across the different tissues (PBMCs and brain temporal cortex). They support a relationship between increased DNAm and decreased *OXTR* gene expression, with the suggestion that there may be sex-specific effects (findings in males and not female, in a small cohort).

A second study by Elagoz Yuksel et al., found the opposite effect in children with ASD, with decreased DNAm in two of four areas of the *OXTR* including MT1 (exon 1) and MT3 (intron 1, exon 2 and intron 2) when peripheral blood samples of 27 children with ASD were compared to 39 healthy controls [60]. Both ASD and healthy control children were predominantly male. It is important to note that these investigators did not find any significant associations within the MT2 area, which had been previously been implicated by Gregory et al. [46] and Kusui et al. [37].

A third study by Rijlaarsdam et al., investigated *OXTR* DNAm associated with the specific SNP *rs 53,576* in cord blood sampled at birth from children later diagnosed with autistic traits at six years’ of age [45]. Increased DNAm was found in those children with *rs53576* G-allele homozygous who demonstrated higher social problem scores. Prenatal maternal stress exposure was also investigated but not found to be associated with *OXTR* DNAm in this sample of children.

### Psychotic disorders

Rubin et al., investigated the whole blood samples of 167 patients with both affective and non-affective psychoses compared to 75 healthy controls [48]. A further component of this study was fMRI of a subsample of this cohort during a facial emotional recognition test. *OXTR* DNAm was examined in one specific CpG site (− 934), around MT2 segment, similar to studies by Jack et al. [50] and Puglia et al. [51]. Findings point to increased DNAm when patients with non-affective psychosis were compared to those with affective psychosis. Gender specific effects were noted with increased DNAm in females with psychosis compared with males, increased DNAm associated with poorer emotional recognition in females and controls than males, and increased DNAm associated with smaller brain volumes in areas related to social cognition (temporal-limbic and pre-frontal regions) in females with non-affective psychosis and healthy control females. This study also measured oxytocin protein levels but found no significant association with these and *OXTR* DNAm in the psychotic or control group unlike the study by Dadd et al., when investigating callous unemotional children [47]. Gender differences were noted when plasma oxytocin levels and DNAm were investigated with a negative association for males (*p* = 0.04) and a positive association for females (*p* = 0.03).

## Discussion

The role of *OXTR* DNAm in human social and emotional functioning is a relatively new area of investigation. There are currently few studies in the field and methods and findings are highly variable, precluding firm conclusions about the role of *OXTR* DNAm. However, within this context, several non-replicated associations between *OXTR* DNAm and outcomes in various domains are emerging in the literature. These include positive associations between *OXTR* DNAm and callous-unemotional traits in youth [47, 58], social cognitive deficits in ASD [45, 46], rigid thinking in anorexia nervosa [49], affect regulation problems [41, 57] and mood deficits [38–40] as well as limbic regions linked with facial and emotional recognition [48, 50, 51]. In contrast, reduced *OXTR* DNAm has been associated within indicators of perinatal stress [55], postnatal depression [56], social anxiety [53] and autism in children [60].

Taken together, findings suggest a role for increased *OXTR* DNAm (which may indicate reduced receptor expression) in general impairments in social, cognitive and emotional functioning, and decreased *OXTR* DNAm (which may indicate increased receptor expression) in specific patterns of impairment related to mood and anxiety disorders (but not all). However, patterns of association both within and across outcomes are difficult to clearly demarcate, and most likely reflect the broad range of differences that exist between studies at this early point in the development of the field. For example, reported associations for mood disorders are in both direction, which may reflect striking differences between studies, including type of mood disorder under investigation, epigenome regions studied, underlying variation in genotypic and hormonal factors [39, 56] as well as the effect of aging [38].

Furthermore, exceedingly little is known about the functional implications of *OXTR* DNAm in humans. Only one study has reported a relationship between increased *OXTR* DNAm and reduced *OXTR* expression [46]. Another has reported a relationship between mean *OXTR* DNAm across eleven probes (spanning exon 1 to intron 1) and circulating oxytocin protein levels; however, this was not replicated in another study examining a single CpG site (− 934) in intron 1 [48]. This is an important area for future research. Genetic association findings are equally non-conclusive with results differing considerably across the studies. Lack of reproducibility is common in genetic association studies and likely reflects small sample sizes of unknown representation with underpowering. Future progress on genetic influences will depend on access to larger samples and benefit by a shift from its current focus on candidate SNPs to genome-wide analysis (GWAS). Furthermore, replication opportunities are essential for establishing the credibility of reported genetic associations. Earliest canvassing of these opportunities would permit optimised alignment of research samples and instrumentation.

A further driver of heterogeneity of DNAm findings is choice of tissue for analysis. Studies in humans are limited to peripheral tissue collections i.e. buccal, blood or saliva. The common embryological origin of brain and buccal cells suggests that these two cell types may have more in common than blood cells, and that using buccal cells as a peripheral biomarker might provide a closer approximation to central brain processes, relative to other cell types [61]. At minimum, the tissue specific nature of many epigenetic processes is likely to be an important contributing factor. The different methods of analysis of *OXTR* DNAm from individual probe methylation to EWAS analysis reflect the challenges of cost, in a new area of investigation which further complicate result comparisons.

From a statistical methods perspective, the majority of studies have investigated methylation at numerous individual CpG sites [38, 40, 41, 45, 46, 49, 52–55, 57, 58], but only a few studies have considered adjustment for multiple comparisons in their analysis, [41, 47–49, 53, 55, 56], to reduce the risk of false positive findings (type 1 errors). This is particularly problematic for studies which have investigated a large number of gene regions and CpG sites. It thus remains possible that at least some of the findings shown here are chance associations, and replication in an independent sample is crucial. On the other hand, some of the studies have used factor analysis to reduce the number of CpG sites and thus tests [40, 45, 47, 58]. The downside of these studies is that factor analysis is a data driven process and does not necessarily make biological sense. Further, conclusions cannot be drawn about the impact of DNA methylation at individual CpGs.

Longitudinal cohort studies beginning in the perinatal period provide the optimal opportunity to study humans at crucial time points during life. This informs knowledge as to how epigenetics contributes to the changing phenotype. Ng et al. identified that in thirty-four life course studies related to the epigenetic mechanism of DNAm, only four studies included information taken from more than 1-time point. Of these four, one study involved epigenetic changes over a relatively short time period (28–180 days) [62]. The Avon Longitudinal study of Parents and Children (ALSPAC) has collected serial samples across the life-span from individual participants providing the gold standard in longitudinal cohort studies for epigenetic analysis [63]. Longitudinal studies which collect biomarkers, at multiple time-points, starting in the perinatal period for epigenetic investigation are unique as an assessment of the changing life-course phenotype. Cross-sectional analyses of epigenetic data lack utility in attempting to understand developmental change or causation direction in epigenetic epidemiology [64].

## Conclusion

This narrative literature review examines findings from nineteen papers, which have investigated *OXTR* DNAm associated with social and emotional functioning in humans. The broad and heterogeneous nature of these studies preclude definitive conclusions. This is typical of a new field of research where the study diversity in design, methodology, phenotype and outcome make meaningful conclusions challenging.

Despite these considerable limitations, emerging evidence points to increased *OXTR* DNAm in general impairments of social, cognitive and emotional functioning, and decreased *OXTR* DNAm in specific patterns of impairment related to mood and anxiety disorders (but not in all). These higher-level patterns are speculative at best in this stage of the field’s early development. The essential “next step” is to establish with greater certainty the functional relationship between *OXTR* DNAm and actual *OXTR* gene expression. Related to this is greater clarity about potential differences in *OXTR* DNAm across peripheral tissues currently collected in human studies in relation to central brain tissue *OXTR* processes. If these fundamentals can be established, progress would be facilitated by increased homogeneity in study design, including phenotypic definition and measurement. This includes greater investment in high quality measurement, such as micro-coding of social interaction using gold-standard protocols, for example, human attachment behaviour, within observational designs [65]. Future progress would also be enhanced by greater investment in embedded DNAm studies within longitudinal designs that enable temporal ordering of relationships with biological collections at multiple time-points. The Developmental Origins of Health and Disease (DOHaD) paradigm emphasises the centrality of exposures within the first 1000 days from conception, however sensitive periods may occur both before and after birth [66]. Longitudinal cohort studies, particularly those with mature cohorts that cross more than one generation, will provide important future opportunities for investigating change over time. Of particular interest are longstanding cohorts prospectively tracking offspring development, given their unique opportunity to explore effects beyond a single generation, to those that transmit to subsequent generations.

## Abbreviations

ADI-R: Autism Diagnostic Interview-revised
ALSPAC: Avon Longitudinal study of parents & children
AQ: Autism-Spectrum Quotient
ASD: Autistic Spectrum Disorder
AUCg: Area under curve with respect to ground
BAI: Beck Anxiety Inventory
BDI: Beck Depression Inventory
BMI: Body Mass Index
BSI: Brief Symptom Inventory
CARS: Childhood Autism Rating Scale
CBCL: Child Behaviour Checklist
CBT: Cognitive Behavioural therapy
CpG island: Cytosine phosphate Guanine dinucleotide island
CTQ: Childhood trauma questionnaire
CU: Callous Unemotional traits
dACC: Dorsal anterior cingulate cortex
DOHaD: Developmental Origins of Health and Disease
DSM IV: Diagnostic and Statistical Manual of Mental Disorders, fourth Edition
DSM IV-TR: Diagnostic and Statistical Manual of Mental Disorders, fourth Edition text revision
EDE-Q: Eating Disorders Examination-Questionnaire
EDPS: Edinburgh Postnatal Depression Scale
ESME: Epigenetic Sequencing Methylation analysis software
EWAS: Epigenome Wide Association Studies
fMRI: Functional magnetic resonance imaging
GWAS: Genome Wide Association Studies
HAMA: Hamilton Anxiety Scale
ILE: Inventory of Life Events
IQ: Intelligence
kb: kilobytes
MDD: Major Depressive Disorder
MMSE: Mini Mental State Examination to screen for cognitive impairments
OCD: Obsessive Compulsive Disorder
OXT: Oxytocin
OXTR: Oxytocin receptor gene
PBI: Parental Bonding Instrument
PDP: Pervasive behaviour checklist
PMCS: Peripheral mononuclear cells in venous blood
SCID: Structured Clinical Interview from DSM IV
SIAS: Social Interaction Anxiety Scale
SNPs: Single nucleotide polymorphisms
SPS: Social Phobia Scale
SRS: Social Responsiveness Scale
STAI: Spielberger State and Trait Anxiety Inventory
TICS-K: Trier Inventory of Chronic Stress – short version
TSST: Trier Social Stress test
Y-BOCS: Yale-Brown Obsessive-Compulsive Scale
YGTTS: Yale Global Tic Severity Scale
